# The effect of demographic characteristics on the relationship between smoking and dry mouth in Iran: a cross-sectional, case-control study

**DOI:** 10.4178/epih.e2021017

**Published:** 2021-02-28

**Authors:** Shahla Kakoei, Amir Hossein Nekouei, Sina Kakooei, Hamid Najafipour

**Affiliations:** 1 Oral and Dental Diseases Research Center, Kerman University of Medical Sciences, Kerman, Iran; 2 Social Determinants of Oral Health Research Center, School of Dentistry, Kerman University of Medical Sciences, Kerman, Iran; 3 Endodontology Research Center, Kerman University of Medical Sciences, Kerman, Iran; 4 Physiology Research Center, Institute of Neuropharmacology, Kerman University of Medical Sciences, Kerman, Iran

**Keywords:** Cigarette smoking, Xerostomia, Demography, Effect modifiers

## Abstract

**OBJECTIVES:**

The effect of age, sex, and other demographic factors on the relationship between smoking and dry mouth remains unknown. The aim of this study was to investigate the effects of demographic characteristics on the relationship between dry mouth, also known as xerostomia, and smoking.

**METHODS:**

This case-control study included 5,640 randomly-selected subjects from the second phase of the Kerman Coronary Artery Disease Risk Factors Study, which observed 10,000 participants from 2014 to 2018. A checklist was used to record the participants’ demographic characteristics and smoking frequency. Each participant completed a six-item Fox questionnaire to measure dry mouth as a dependent variable. The interaction terms of daily cigarette smoking with sex, age, educational level, and marital status were entered into the model. Non-significant terms were removed using hierarchical model selection.

**RESULTS:**

Of the sample, 3,429 (60.8%) did not have dry mouth and were analyzed as controls, whereas 2,211 (39.2%) had xerostomia and were deemed to be cases. Smokers were more likely to have dry mouth in all ages and both sexes (p<0.001). As male became older, the chance of having dry mouth increased more rapidly than among female smokers (p<0.001). In addition, female smokers were more likely to have dry mouth than male smokers (p<0.001).

**CONCLUSIONS:**

The likelihood of dry mouth among daily smokers depended on age and sex. Female smokers were more likely to have dry mouth, and its likelihood increased with age in daily smokers of both sexes, though more rapidly in males.

## INTRODUCTION

Cigarette smoking as a form of tobacco consumption is a serious problem for both public and individual health. According to a World Health Organization (WHO) report, about 5 million adults aged 30 years or older died globally as a result of tobacco consumption in 2004 [1]. In the last 2 decades, there has been an increased interest in exploring the negative impacts of smoking on oral health. Reibel [2] compared various risk factors related to oral health problems in their review.

The likelihood of someone being a smoker depends on demographic characteristics such as age and sex. According to a different WHO report, tobacco smoking is more prevalent among males than females, both globally and in most individual countries [3]. In Iran, 10.3% of males were daily tobacco smokers compared to only 0.3% of females [1]. It has been shown that the prevalence of smoking is associated with demographic factors such as marital status, educational level, and sex [4,5].

A substantial effect of smoking on oral health is the development of dry mouth, a condition characterized by a low salivary flow rate, which can lead to increased plaque, tooth decay, and mouth sores [6,7]. In Kerman, Iran, 55% of people referred to medical and dental clinics experienced dry mouth.

Some evidence supports that the likelihood of dry mouth depends on age and sex [8-10]. Some studies have reported that dry mouth was more prevalent in female than male [8-12]. Furthermore, a study on the prevalence of dry mouth in Australian adults showed that male aged 55-74 years old experienced dry mouth less than female aged 55-74 years old, and this disparity was consistent across age groups [13].

Many studies have also investigated smoking as a cause of dry mouth. Thomson et al. [14] were the first to investigate the relationship between smoking and dry mouth among elderly people by measuring salivary flow rate and using the Shortened Xerostomia Inventory. The effect of long-term tobacco smoking on dry mouth in 20-year-olds to 30-year-olds was evaluated by Khan et al. [15]. In a similar study, Fenoll-Palomares et al. [16], investigated the effect of smoking on unstimulated salivary flow rate in subjects above and below 44 years of age. Rad et al. [7] examined salivary flow rate in long-term smokers compared with that of non-smokers. A similar study was conducted by Dyasanoor & Saddu [6] and Petrušić et al. [17]. Villa & Abati [18] evaluated and compared the prevalence of self-reported dry mouth between current smokers and non-smokers.

In terms of public health, the relationship between dry mouth and smoking in subpopulations is of great importance. To the best of our knowledge, there is no study on the relationship between smoking and dry mouth according to age, sex, educational level, and marital status. Few studies have investigated the relationship between oral diseases and smoking. Hence, the present study was conducted to evaluate the relationship between oral diseases and demographic characteristics in smokers.

## MATERIALS AND METHODS

This case-control, cross-sectional study was conducted on a subsample of 10,000 persons who entered the second phase of the Kerman Coronary Artery Disease Cohort Risk Factors Study (KERCADRS) from September 2014 to December 2018. This study included 5,640 people who were randomly selected from 10,000 participants of KERCADRS.

Demographic variables such as age, sex, educational level, and marital status were measured using a checklist. In addition, three questions were included to determine daily cigarette use (“Have you smoked a cigarette recently?”, “Do you smoke every day?”, “Have you smoked daily in the past?”). People who smoked recently were considered daily smokers, and people who smoked daily in the past were excluded from the study. Current daily smokers were analyzed because they had sufficient exposure to develop dry mouth. In order to measure dry mouth, a Persian version of the 11-item Fox questionnaire was used (“Does your mouth feel dry at night or on awakening?”, “Does your mouth feel dry at other times of the day?”, “Do you keep a glass of water by your bed?”, “Do you sip liquids to aid in swallowing dry foods?”, “Does your mouth feel dry when eating a meal?”, “Do you have difficulties swallowing any foods?”, “Do you chew gum daily to relieve oral dryness?”, “Do you use hard candies or mints daily to relieve oral dryness?”, “Does the amount of saliva in your mouth seem to be: too little, too much, or you do not notice it?”) [19]. For this study, least 1 positive answer to the 6 starred questions indicated dry mouth [20]. Individuals who had dry mouth were considered as cases and the others were considered as controls.

### Statistical analysis

Data were analyzed using SPSS version 25 (IBM Corp., Armonk, NY, USA). The relationship between daily cigarette smoking, dry mouth, and demographic factors such as sex, age, educational level, and marital status was investigated using univariate analysis. A multivariable analysis was performed using a multiple logistic regression model. Self-reported dry mouth was considered as the dependent variable and the interaction terms of daily cigarette smoking with sex, age, educational level, and marital status were entered into the model, with non-significant terms being removed by the hierarchical model selection. The goodness of fit of the statistical model and its terms were evaluated at a significance level of 5%.

### Ethics statement

The Ethics Committee of Kerman University of Medical Sciences approved the protocol of this study (ethical code: 93/310KA). The research process and its objectives were explained to the participants, and informed consent forms were signed by the subject or the subject’s parents/legally authorized representative before starting the project. The questionnaires were anonymous, and the subjects were assured of the confidentiality of the data.

## RESULTS

A total number of 5,639 people participated in this study. Of this number, 3,429 (60.8%) were considered controls and did not experience dry mouth, while 2,211 (39.2%) were considered cases and did experience dry mouth. A comparative evaluation of demographic variables between cases and controls is shown p=0.002 in Table 1. The rate of dry mouth was significantly higher in married participants (p=0.002) and illiterate participants (p<0.001). The age of participants considered cases was on average significantly higher than the age of those considered controls. The prevalence of daily smoking was also higher among male and married participants (p<0.001). Moreover, the average age of daily smokers was higher than those of non-daily smokers (p<0.001). Therefore, these variables met the criteria to be considered potential effect modifiers. Although educational level was not significantly different between daily and non-daily smokers (p=0.334), we still considered this variable as a potential effect modifier in our multiple logistic regression model.

A hierarchical model selection process was used to select interaction terms in the final model. The Hosmer-Lemeshow test showed that the model had good fit (p=0.288). The estimated coefficients of logistic regression are presented in Table 2. Of all the 2-way interactions, the interaction of sex and age with daily smoking was the most significant (p<0.05). The results of this test showed that the relationship between daily smoking and dry mouth was influenced by sex and age. It was also shown that married people had a higher likelihood of experiencing dry mouth than unmarried people but the difference was not significant. And people with higher educational levels had a lower likelihood of experiencing dry mouth (p<0.001). Nevertheless, these variables had no effect on the relationship between dry mouth and daily cigarette smoking. For simplicity, odds ratios were calculated based on the model, the results of which are presented in Table 3. These odds ratios show that smokers were more likely to have dry mouth in all ages for both sexes than non-daily smokers. In addition, female daily smokers had a higher likelihood of experiencing dry mouth than male smokers.

## DISCUSSION

The most remarkable finding of this study is that age and sex were determinant factors for the likelihood of experiencing dry mouth among daily smokers. Daily smoking placed females at a greater risk of dry mouth than was the case for males. These results are important because it is necessary to know which groups are most at risk in order to prevent dry mouth and its consequences through smoking cessation programs.

In this study, the prevalence of dry mouth was 39.2%. The prevalence of dry mouth ranged from 0.01% to 45.00% in a different systematic review [21]. Another systematic review [22] of daily smokers reported a range of 0.9% to 64.8%. The prevalence of dry mouth using the Fox questionnaire in our 2013 study was 55% [20]. Based on the overall results, the most salient difference between these studies was the ratio of age and sex among participants.

This study is one of the first studies to observe the relationship between smoking and dry mouth in people of different ages and sexes. Based on the research we have done so far, there is no study with which to compare the results directly (Table 3). From an epidemiological point of view, the strength of the relationship between smoking and dry mouth in subpopulations is very important. The results of our study indicate that the relationship varies substantially within subpopulations. Most studies on the relationship between smoking and dry mouth are observational. Consequently, the sex and age of participants can affect the results of these studies.

Our results showed that people with higher educational levels were less likely to have dry mouth. Socioeconomic inequality is a significant factor regarding differences in the health status of individuals and disease patterns. A likely explanation for this finding is that people with higher levels of education are less exposed to the risk factors for dry mouth due to a greater awareness of hygienic practices [23]. However, the relationship between smoking and xerostomia remained unchanged across educational levels. In addition, our results showed that marital status had no relationship with xerostomia, meaning that single and married people have an equal chance of experiencing dry mouth depending on their smoking status.

In this study, the likelihood of dry mouth increased in smokers and non-smokers with age, which is consistent with the results of other studies [11,12,17,18,24]. This result can be attributed to the fact that salivary secretion decreases as age increases in humans.

Several studies have demonstrated that dry mouth is more prevalent in females [11,12,17,18,25]. It has also been shown that parotid and submandibular gland sizes and flow rates differ between the sexes [26]. The symptoms and effects of dry mouth depend on these flow rates [27]. It has also been shown that smoking has an adverse effect on the quantity and quality of an individual’s saliva [18,28,29]. Therefore, the evidence supports that female smokers have a higher risk of developing dry mouth, as shown by the results of the present study with regard to female daily smokers and female non-smokers. It was also shown that dry mouth was highly prevalent in female daily smokers, which is an important finding for promoting public health.

There are some discrepancies in the results of studies on the effects of smoking on dry mouth. In this regard, it is worth mentioning that other studies defined smokers and frequency of smoking differently. Thomson et al. [25] found that the unstimulated salivary flow rate was higher among cigarette smokers. In that study, smokers were considered people who smoked one or more cigarettes in the last month. They also used a dry mouth inventory in order to measure dry mouth and found that the questionnaire results did not differ between cigarette smokers and non-smokers, which is inconsistent with the results of the present study. This can be attributed to the difference in the definition of smokers. In a similar study, stimulated and unstimulated salivary flow rates were higher in long-term smokers of tobacco than in non-tobacco users. Subjects were considered long-term smokers if they had smoked tobacco for 5-7 years. However, the mean age of participants was 20-30 years in that study [15]. Villa & Abati [18] indicated that there was no difference in the prevalence of self-reported dry mouth between current smokers and non-smokers. Fenoll-Palomares et al. [16] found that there was no difference in the salivary flow rate between smokers and non-smokers. They defined smokers as individuals who smoked without considering the number of cigarettes. Petrušić et al. [17], whose definition of “smoker” is not clear, also indicated that there was no difference in salivary flow rate between smokers and non-smokers, but in smokers, the salivary flow rate was negatively correlated with age. The results of a study by Rad et al. [7] showed that salivary flow rate was lower in long-term smokers, which is consistent with the results reported by Dyasanoor & Saddu [6]. In both studies, smokers were considered subjects who have smoked daily for at least the previous six months, which is consistent with the results of this study.

The main limitation of this study is that it used subjective criteria to measure dry mouth. Some elderly participants, for example, may have had lower education levels, making them unable to fill out the questionnaire by themselves. Even so, it has been shown that the Fox inventory still meets acceptable sensitivity and specificity requirements [5,11] compared to the measure of salivary flow rate. The main strength of this study is that the cases and controls were randomly selected from the same population, thus preventing selection bias. It is also likely that the effect of daily smoking on dry mouth was underestimated, since people who did not smoke daily or used other forms of tobacco were included in the analysis.

Future studies have been suggested to investigate the relationship between smoking and dry mouth using different definitions of smoking as well as different types of smoking. It is also important to highlight the role of age, sex, and smoking habits of participants in the results of future studies. This means that any imbalance in age, sex, and smoking habits of participants can produce biased results. We also suggest that this study be repeated using both subjective questionnaires for measuring dry mouth, such as the Xerostomia Inventory, as well as objective tests.

We conclude that age, sex, and daily cigarette smoking habited affect the likelihood of experiencing dry mouth in a very complex way. In addition, the relationship between dry mouth and smoking differed between male and female of different ages. Therefore, demographic characteristics can affect this relationship.

## Figures and Tables

**Table 1.** Comparison of demographic variables of smokers and non-smokers, and people with dry mouth and without dry mouth

Variables	Daily cigarette smoking (exposure)		p-value	Dry mouth (disease)		p-value
	Yes	No		Did not have	Had	
Sex						
Female	3,388 (99.3)	25 (0.7)	<0.001^1^	2,058 (60.3)	1,354 (39.7)	0.349^1^
Male	1,752 (78.7)	475 (21.3)		1,371 (61.6)	857 (38.4)	
Marital status						
Single	655 (95.1)	34 (4.9)	<0.001^1^	447 (65.0)	241 (35.0)	0.002^1^
Married	4,122 (90.1)	455 (9.9)		2,794 (61.0)	1,783 (39.0)	
Education						
Illiterate	478 (92.5)	39 (7.5)	0.334^1^	256 (49.5)	261 (50.5)	<0.001^1^
School	3,752 (91.1)	368 (8.9)		2,505 (60.8)	1,614 (39.2)	
University	910 (90.7)	93 (9.3)		668 (66.6)	335 (33.4)	
Age (yr)	46.87±0.42	52.87±1.15	<0.001^2^	45.95±0.51	49.44±0.65	<0.001^2^

Values are presented as number (%) or mean±standard deviation.

1 Chi-square test.

2 Independent t-test.

**Table 2.** Results of multivariable logistic regression^1^

Parameter	B	Standard error	p-value
(Intercept)	-0.641	0.199	<0.001
Male	0.572	0.200	0.002
Female		Reference^2^	
Daily smoker	0.828	2.126	0.348
Non-daily smoker		Reference^2^	
Married	0.11	0.101	0.138
Single		Reference^2^	
University	-0.278	0.115	0.007
School	-0.509	0.132	<0.001
Illiterate		Reference^2^	
Age	0.009	0.003	0.001
Daily smoker * Age	0.001	0.037	0.489
Sex * Daily smoker	0.572	0.200	0.002
Sex * Age	-0.012	0.004	0.001
Age * Daily smoker * Sex	0.006	0.003	0.022

1 Dependent variable: dry mouth.

2 Reference category: don’t have dry mouth.

**Table 3.** Odds ratios of having dry mouth by age, sex, and smoking status

Characteristics		Age (yr)		
		20	40	60
Male	Daily smoker vs. not daily smoker	2.64 (1.97, 3.54)	3.03 (2.39, 3.83)	3.49 (2.40, 5.06)
Female	Daily smoker vs. not daily smoker	2.33 (1.67, 3.25)	2.38 (1.84, 3.07)	2.43 (1.20, 4.92)
Daily smoker	Female vs. male	1.57 (1.10, 2.23)	1.39 (1.14, 1.69)	1.23 (1.07, 1.41)

Values are presented as odds ratio (95% confidence interval).
